# Physical activity is related to quality of life in older adults

**DOI:** 10.1186/1477-7525-4-37

**Published:** 2006-06-30

**Authors:** Luke S Acree, Jessica Longfors, Anette S Fjeldstad, Cecilie Fjeldstad, Bob Schank, Kevin J Nickel, Polly S Montgomery, Andrew W Gardner

**Affiliations:** 1 Department of Health and Exercise Science, University of Oklahoma, Norman, OK, USA; 2 CMRI Metabolic Research Center, University of Oklahoma Health Sciences Center, 1122 N.E. 13^th ^Street, ORI-W 1400, Oklahoma City, OK 73117, USA

## Abstract

**Background:**

Physical activity is associated with health-related quality of life (HRQL) in clinical populations, but less is known whether this relationship exists in older men and women who are healthy. Thus, this study determined if physical activity was related to HRQL in apparently healthy, older subjects.

**Methods:**

Measures were obtained from 112 male and female volunteers (70 ± 8 years, mean ± SD) recruited from media advertisements and flyers around the Norman, Oklahoma area. Data was collected using a medical history questionnaire, HRQL from the Medical Outcomes Survey short form-36 questionnaire, and physical activity level from the Johnson Space Center physical activity scale. Subjects were separated into either a higher physically active group (n = 62) or a lower physically active group (n = 50) according to the physical activity scale.

**Results:**

The HRQL scores in all eight domains were significantly higher (p < 0.05) in the group reporting higher physical activity. Additionally, the more active group had fewer females (44% vs. 72%, p = 0.033), and lower prevalence of hypertension (39% vs. 60%, p = 0.041) than the low active group. After adjusting for gender and hypertension, the more active group had higher values in the following five HRQL domains: physical function (82 ± 20 vs. 68 ± 21, p = 0.029), role-physical (83 ± 34 vs. 61 ± 36, p = 0.022), bodily pain (83 ± 22 vs. 66 ± 23, p = 0.001), vitality (74 ± 15 vs. 59 ± 16, p = 0.001), and social functioning (92 ± 18 vs. 83 ± 19, p = 0.040). General health, role-emotional, and mental health were not significantly different (p > 0.05) between the two groups.

**Conclusion:**

Healthy older adults who regularly participated in physical activity of at least moderate intensity for more than one hour per week had higher HRQL measures in both physical and mental domains than those who were less physically active. Therefore, incorporating more physical activity into the lifestyles of sedentary or slightly active older individuals may improve their HRQL.

## Background

Successful aging encompasses multiple dimensions of health, including physical, functional, social, and psychological well-being [1]. Maintaining a high level of quality of life into advanced age is a growing public health concern as the older adult population continues to increase. In fact, one of the primary goals of Healthy People 2010 is to improve both the quality and the number of years of healthy life [2]. Quality of life is frequently measured in investigations to evaluate the health of both clinical and general populations [3], and is therefore termed health-related quality of life (HRQL). Core dimensions in a HRQL assessment include physical and social functioning, emotional well-being, role activities, and individual health perceptions [3,4].

Physical activity has a beneficial effect on HRQL in patients with depression [5], intermittent claudication [6], coronary disease [7], and multiple organ dysfunctions [8]. In 2001, a review concluded that physical activity, often in the form of endurance and/or resistance training exercise, was positively associated with HRQL, regardless of age, health and activity status [3]. Data from the 2001 Behavioral Risk Factor Surveillance System, consisting of a large sample with a wide range of demographic and physical characteristics, found that people attaining the recommended amounts of physical activity had higher HRQL than their less active counterparts [9]. However, few of these studies have addressed the relationship between physical activity and all domains of HRQL in healthy, older adults.

In aging populations, the health benefits from physical activity (i.e., decreased risk for cardiovascular disease, diabetes, hypertension, cancer, and all-cause mortality) are well-known [10-12]. However, it is not clear whether physical activity improves specific domains of HRQL. A recent report found that physical activity was associated with less bodily pain in a carefully selected group of sedentary older adults who had either high normal blood pressure or mild hypertension, but who were free of clinical manifestations of chronic diseases [13]. The lack of association between physical activity and the other domains of HRQL may have occurred due to the narrow range in physical activity level of this sedentary cohort. Thus, studying older adults who are physically active in addition to those who are sedentary may be necessary to delineate the association between physical activity and HRQL.

The main purpose of this study was to determine if physical activity was associated with HRQL in apparently healthy, older subjects.

## Methods

### Subjects

#### Recruitment

A total of 112 subjects (63 females and 49 males) between the ages of 60 and 89 years were recruited from newspaper advertisements and media flyers posted around the Norman, OK area. Prior to investigation, each subject completed a written informed consent. The study was conducted with the approval of the Institutional Review Board at the University of Oklahoma.

#### Inclusion and exclusion criteria

Men and women who were 60 years of age and older were included in this study. Participants with a history of overt cardiovascular disease (i.e., myocardial infarction, stroke, congestive heart failure, lower extremity revascularization, and peripheral arterial disease confirmed by an ankle/brachial index < 0.90) or chronic obstructive pulmonary disease were excluded because of the possible confounding influences that cardiovascular diseases may have on both physical activity and HRQL.

### Measurements

#### Demographic measures and medical history

A detailed, medical history was obtained from each participant. The medical history addressed all of the aforementioned exclusion criteria, as well as other comorbid conditions such as hyperlipidemia, dyspnea, diabetes, arthritis, hypertension. Height was measured using a stadiometer, with the measurement taken to the nearest 0.1 cm. Weight was measured using a balance scale, with the measurement taken to the nearest 0.1 kg. Height and weight measurements were then used to calculate body mass index (BMI): [BMI = (weight in kilograms)/(height in meters)^2^]. Waist-to-hip ratio was measured with a Gulick measuring tape, with a horizontal measure at both the waist and hip sites, taken to the nearest 0.1 cm. Blood pressure and heart rate were measured with a Critikon automated Dinamap sphygmomanometer following 10 minutes of supine rest, and ankle/brachial index was obtained by Doppler ultrasound [6].

#### Physical activity

Subjects were divided into either a lower or higher physical activity group based on their score on the Johnson Space Center physical activity scale, as previously described [14]. This scale consists of the following scores: 0 = avoid physical activities, 1 = light physical activities done occasionally, 2 = moderate physical activities done regularly for less than one hour per week, 3 = moderate physical activities done regularly for more than one hour per week, 4 = heavy physical activities done regularly for less than 30 minutes per week, 5 = heavy physical activities done regularly between 30 and 60 minutes per week, 6 = heavy physical activities done regularly between 1 and 3 hours per week, and 7 = heavy physical activities done regularly for more than 3 hours per week. Subjects were asked to select the number that best corresponded to their physical activity level during the previous month. Subjects who had a score of 2 or below were included in the lower physical activity group (n = 50), whereas subjects who had a score of 3 or above were included in the higher physical activity group (n = 62). In this likert scale, those subjects who scored less than 3 were far below the minimum recommendation of physical activity from the Center for Disease Control and the American College of Sports Medicine [15], whereas those who scored 3 or higher were closer to meeting this recommendation or exceeded it. The Johnson Space Center physical activity scale is a valid measure of peak oxygen uptake [14] and monitored physical activity (r = 0.44, p < 0.001) in older adults [16]. Furthermore, the prediction of peak oxygen uptake by using the Johnson Space Center physical activity scale has been validated in gender-specific models, as well as in a generalized model which included both men and women [17].

#### Health-related QoL

The Medical Outcomes Survey Short Form-36 (MOS SF-36) questionnaire was administered to assess HRQL over the previous four weeks [18]. The MOS SF-36 is a widely used, reliable, and valid criterion measure of HRQL in numerous populations [3]. The MOS SF-36 questionnaire has 36 questions that are scored to measure eight domains of HRQL pertaining to both physical and mental health [19,20]. The domains of physical functioning, role limitations due to physical health (role-physical), bodily pain, and general health comprised the physical component of HRQL, whereas the domains of vitality, social functioning, role limitations due to emotional health (role-emotional), and mental health comprised the mental component of HRQL [19]. Each domain was scored using a scale ranging between 0 and 100, with higher scores indicating a higher HRQL than lower scores. Internal consistency of the MOS SF-36 is good, with Cronbach's alpha ranging from 0.76 to 0.90 for all domains of the questionnaire [21].

### Statistical analysis

SPSS version 11.5 for Windows was used to analyze all data. Unpaired t-tests were performed on continuous variables to determine differences in HRQL and demographic measurements between the high active and low active groups. Chi-square tests were performed on categorical variables to determine differences between the two groups in the prevalence of comorbid conditions obtained from the medical history. Analysis of covariance was performed to determine whether differences in HRQL measurements between the high active and low active groups persisted after adjusting for group differences in demographic and comorbid measures. Statistical significance was set at P = 0.05. Measurements are reported as mean ± standard deviation (SD).

## Results

The baseline characteristics of the subjects in the two physical activity groups are displayed in Table 1. All values were similar (p > 0.05) between the two groups, except for the gender composition and the prevalence of hypertension. The group reporting lower physical activity had a greater proportion of females (p = 0.033) and a greater prevalence of hypertension (p = 0.041) than the group reporting higher physical activity. Both groups consisted largely of Caucasian subjects (p > 0.05).

**Table 1:** Subject characteristics between those who have lower and higher levels of physical activity. Values are means (SD) or percentages.

Variables	Lower Physical Activity Group (n = 50)	Higher Physical Activity Group (n = 62)	Test Statistic*	P Value
Age (yrs)	70 (8)	69 (8)	0.36	0.717
Weight (kg)	76.8 (14.8)	78.1 (14.4)	0.49	0.626
Body Mass Index (kg/m^2^)	28.6 (5.6)	27.2 (4.9)	1.00	0.320
Waist-to-Hip Ratio	0.84 (0.09)	0.87 (0.10)	1.25	0.215
Gender (% Female)	72	44	4.55	0.033
Race (% Causasian)	92	94	1.46	0.227
Hypertension (%)	60	39	4.18	0.041
Hyperlipidemia (%)	34	32	< 0.01	0.979
Diabetes (%)	14	13	< 0.01	0.963
Dyspnea (%)	36	23	1.39	0.238
Arthritis (%)	68	53	1.42	0.234

* t-scores and chi-square scores.

All eight domains of HRQL were higher (p < 0.05) in the high active group than the low active group (Table 2). After adjusting for gender and hypertension (Table 3), the following five domains of HRQL remained higher in the high active group: physical functioning (p = 0.029), role-physical (p = 0.022), vitality (p = 0.001), social functioning (p = 0.04) and bodily pain (0.001). The domains of role limitations due to emotional health, mental health, and general health were no longer different (p > 0.05) between the two activity groups after adjusting for gender and hypertension.

**Table 2:** Health-related quality of life measures in subjects who have lower and higher levels of physical activity. Values are means (SD).

Variables	Lower Physical Activity Group (n = 50)	Higher Physical Activity Group (n = 62)	Test Statistic*	P Value
Physical Function	65 (24)	83 (16)	2.94	0.004
Role-Physical	60 (39)	85 (25)	2.75	0.007
Role-Emotional	71 (36)	92 (24)	2.29	0.024
Vitality	56 (19)	76 (14)	3.38	< 0.001
Mental Health	77 (16)	84 (12)	2.17	0.032
Social Function	81 (18)	93 (16)	2.08	0.040
Bodily Pain	64 (26)	84 (16)	3.17	0.002
General Health	62 (18)	75 (15)	2.47	0.015

* t-scores.

**Table 3:** Adjusted health-related quality of life measures in subjects who have lower and higher levels of physical activity. Values are means (SD).

Variables	Lower Physical Activity Group (n = 50)	Higher Physical Activity Group (n = 62)	Test Statistic*	P Value
Physical Function	68 (21)	82 (20)	4.90	0.029
Role-Physical	61 (36)	83 (34)	5.40	0.022
Role-Emotional	75 (33)	89 (31)	3.17	0.078
Vitality	59 (16)	74 (15)	11.44	< 0.001
Mental Health	78 (14)	83 (13)	2.72	0.102
Social Function	83 (19)	92 (18)	4.32	0.040
Bodily Pain	66 (23)	83 (22)	11.44	< 0.001
General Health	65 (16)	73 (15)	2.61	0.109

Values were adjusted for gender and hypertension. * t-scores.

## Discussion

The primary findings of this investigation were that healthy older adults who participated in regular physical activity of at least moderate intensity for more than one hour per week had higher values in all eight domains of HRQL than those who were less physically active. After adjustment for gender and hypertension, the group reporting higher physical activity had higher values in five of the domains of HRQL than the group with lower physical activity.

Several studies have shown that organized, high-intensity exercise regimens can benefit HRQL in both diseased [3,5,22-24] and healthy populations [25,26]. Our study extends these findings by showing that the less-structured and less-intense nature of physical activity is positively related to multiple domains of HRQL in healthy, older adults. An active lifestyle preserves physical function in older adults [27], which may possibly contribute to higher levels of HRQL scores in domains related to physical health. In a carefully selected group of sedentary older adults, habitual physical activity level was associated with less bodily pain, but not with the other domains of HRQL [13]. It is possible that the range in physical activity level was too narrow within the sedentary older adults, thereby limiting the influence that physical activity may have exerted on HRQL domains. The present study supports this notion, as the group having higher physical activity levels had greater values in all of the domains of HRQL related to physical health (i.e., physical function, role limitations due to physical health, bodily pain, and general health) than their more sedentary counterparts.

After adjusting for group differences in gender composition as well as the prevalence of hypertension, all of the HRQL measures in the physical health domain remained higher in the more physically active group except for the measure of general health. This suggests that the HRQL domains of physical function, role limitations due to physical health, and bodily pain are positively associated with physical activity, independent of hypertension and gender. In contrast, group differences in hypertension and gender explained the lower scores in general health in those with lower physical activity. The lack of an independent relationship between physical activity level and the domain of general health agrees with the observation in sedentary older adults [13], but contradicts other reports that found improvements in perceived health status following an exercise program [28,29].

In addition to the physical health measures of HRQL, a physically active lifestyle is positively associated with components of mental health in older adults as well [3]. The present investigation found that the group with higher physical activity levels had higher values in all of the domains of HRQL related to mental health (i.e., vitality, social functioning, role limitations due to emotional health, and mental health) than their more sedentary counterparts. After adjusting for group differences in gender composition as well as the prevalence of hypertension, the mental health domains of vitality and social functioning remained higher in the more physically active group, whereas the role-emotional and mental health domains were no longer different between the two groups. Thus, higher levels of physical activity are related to higher scores of vitality and social functioning independent of hypertension and gender. The association between physical activity on mental health domains of vitality and social functioning may be mediated through differences in maximal oxygen uptake and in body fat percentage [13].

The lack of an independent relationship between physical activity level and the domains of mental health and role limitations due to emotional health found in the present investigation is supported by previous studies [13,26,30]. Collectively, these results suggest that physical activity has minimal impact on mental health and role limitations due to emotional health. However, our findings show that hypertension and gender have a more influential role on these HRQL domains, which agree with previous reports that individuals with hypertension have lower values of HRQL in both a general population [31], and in cardiac patients [32]. It is possible that individuals who have been diagnosed with hypertension have a greater awareness through medical counseling about the increased cardiovascular health risk associated with poorly controlled blood pressure, resulting in concern about their health status. Less is known about the influence of gender on HRQL. Repetto et al. reported that, as a result of a longer life expectancy, women are more apt to live alone and to receive assistance from others for daily life activities [33], which may influence their perception of HRQL. In addition to these findings, the present study emphasizes the need to more clearly define the independent role that hypertension and gender have on HRQL.

The main limitation of this study is the utilization of a cross-sectional design, which does not allow a true causal relationship to be established. Although physical activity may affect HRQL, it is also possible that HRQL has an impact on physical activity. It should be noted that our recruitment methods were biased toward older individuals who responded to newspaper advertisements and flyers posted in public facilities. Additionally, the Johnson Space Center questionnaire determines an overall level of physical activity without quantifying various types of physical activity. However, the questionnaire does contain questions about sweating that coincide with light, moderate, and heavy activity, thereby increasing the likelihood for more accurate reporting. Another limitation is that both physical activity and HRQL measures were obtained by self-report. However, this limitation is minimized because both instruments are valid and reliable [14,18], and are simple for health professionals to administer. Finally, the current study used a relatively small convenience sample of healthy older adults who had few co-morbid conditions. Consequently, the findings of this study cannot be generalized to older adults who have more serious health conditions.

## Conclusion

In summary, healthy older adults who participated in regular physical activity of at least moderate intensity for more than one hour per week had higher values in all eight domains of HRQL than those who were less physically active. After adjustment for gender and hypertension, the group reporting higher physical activity had higher values in five of the domains of HRQL than the group with lower activity. These findings give added strength to previous observations that higher levels of physical activity may improve HRQL [3,9,13]. Therefore, incorporating more than one hour of moderate-intensity physical activity each week into the lifestyles of older individuals who are either sedentary or slightly active may improve their HRQL.

## Competing interests

The author(s) declare that they have no competing interests.

## Authors' contributions

LSA acquired data, assisted with statistical analyses and their interpretation, and drafted and revised the manuscript. JL conceived and designed the study, acquired data, performed statistical analyses, and drafted the manuscript. BS and PSM recruited subjects and revised the manuscript. ASF, CF, KJN acquired data and revised the manuscript. AWG conceived and designed the study, assisted with statistical analyses and their interpretation, and drafted and revised the manuscript. All authors read and approved the final manuscript.
